# Chronic Inflammation in Asthma: Looking Beyond the Th2 Cell

**DOI:** 10.1111/imr.70010

**Published:** 2025-02-27

**Authors:** Simone E. M. Olsthoorn, Anneloes van Krimpen, Rudi W. Hendriks, Ralph Stadhouders

**Affiliations:** ^1^ Department of Pulmonary Medicine Erasmus MC University Medical Center Rotterdam the Netherlands

**Keywords:** asthma, cytokines, ILC2, Tc2 cell, Th2 cell, type 2 inflammation

## Abstract

Asthma is a common chronic inflammatory disease of the airways. A substantial number of patients present with severe and therapy‐resistant asthma, for which the underlying biological mechanisms remain poorly understood. In most asthma patients, airway inflammation is characterized by chronic activation of type 2 immunity. CD4^+^ T helper 2 (Th2) cells are the canonical producers of the cytokines that fuel type 2 inflammation: interleukin (IL)‐4, IL‐5, IL‐9, and IL‐13. However, more recent findings have shown that other lymphocyte subsets, in particular group 2 innate lymphoid cells (ILC2s) and type 2 CD8^+^ cytotoxic T (Tc2) cells, can also produce large amounts of type 2 cytokines. Importantly, a substantial number of severe therapy‐resistant asthma patients present with chronic type 2 inflammation, despite the high sensitivity of Th2 cells for suppression by corticosteroids—the mainstay drugs for asthma. Emerging evidence indicates that ILC2s and Tc2 cells are more abundant in severe asthma patients and can adopt corticosteroid‐resistance states. Moreover, many severe asthma patients do not present with overt type 2 airway inflammation, implicating non‐type 2 immunity as a driver of disease. In this review, we will discuss asthma pathophysiology and focus on the roles played by ILC2s, Tc2 cells, and non‐type 2 lymphocytes, placing special emphasis on severe disease forms.

## 1 Introduction

### 1.1 Asthma

Asthma is a non‐communicable chronic inflammatory disease that affects the airways. In asthma patients, a complex interplay of various types of immune cells and lung structural cells together causes bronchial hyper‐reactivity, excessive mucus production, and airway narrowing. These symptoms can in turn lead to repeated periods of wheezing, shortness of breath, and tightness of the chest [1]. Asthma is a very common disorder, with an estimated 300 million people affected worldwide, and these numbers continue to grow. Given its prevalence and chronic nature, asthma poses a significant societal burden in both morbidity and healthcare spending. In 2013, the total annual medical cost of asthma treatment was estimated at $50.3 billion in the United States [2], with projections for the period 2019–2038 indicating a substantial increase in costs [3]. Asthma symptoms can be effectively controlled in many patients using a combination of inhaled anti‐inflammatory corticosteroids and a β2‐adrenergic agonist for bronchodilation [1, 4, 5]. However, 5%–10% of patients present with a form of therapy‐resistant disease that is often accompanied by frequent exacerbations triggered by respiratory viral infections [6, 7, 8]. Importantly, these patients are frequently treated with multiple courses of high‐dose systemic corticosteroids, which is associated with serious adverse effects [9]. It is this group of treatment refractory patients that suffers the highest disease burden and accounts for over half of the asthma‐associated healthcare expenditure, with 80% of the total direct costs of asthma attributed to the treatment of exacerbations [7, 10]. A major goal of current asthma research is to better understand the underlying causes of therapy resistance and exacerbations, to ultimately provide better treatment options to this group of asthma patients, improve their quality of life, and reduce the economic impact of asthma.

Why certain individuals are more susceptible to develop asthma remains an open question and a topic of active investigation. Environmental exposures, particularly in early life, appear critically important in the development of asthma [11]. These include risk factors such as antibiotics treatment during the first year of life or frequent respiratory viral infections, but also protective factors such as exposure to endotoxins (e.g., growing up on a farm) [12, 13, 14]. In addition, genetic differences are well‐described to modify asthma susceptibility. Twin studies have previously indicated that as much as 70% of asthma susceptibility is explained by genetic factors [15, 16]. Genome‐wide association studies (GWAS) involving thousands of individuals have consistently identified > 100 genetic loci carrying asthma‐associated variants [17, 18], which have been used to construct genetic risk scores that can identify individuals with an elevated risk of developing asthma [19].

Apart from their response to therapy, asthma patients can show substantial differences in disease onset and clinical presentation. Asthma often starts during childhood (childhood‐onset asthma) but can also develop during adulthood (adult‐onset asthma), with the latter group displaying the highest overall symptom burden and exacerbation frequencies [1]. In terms of underlying disease pathophysiology, asthma has historically been divided into two subtypes: allergic and non‐allergic (or “intrinsic”) asthma. Around half of the adult patients and most children have allergic asthma, which is characterized by an adaptive type 2 immune response (*see below*) against harmless allergens such as house dust mite (HDM), animal dander, or pollen, but also against specific occupational allergens [1, 20, 21]. What drives non‐allergic asthma is still unclear, although it does not appear to involve allergen‐specific immune responses. Recently, high‐dimensional and more unbiased analyses have revealed a spectrum of asthma phenotypes (often referred to as “endotypes”) that goes beyond a simple division of patients based on age of onset or allergy status [22, 23]. Currently, asthma patients are classified into type 2‐high or type 2‐low phenotypes based on the involvement of a type 2 inflammatory response, which can be further subdivided using additional characteristics such as allergy status, age of onset, inflammatory parameters (i.e., high levels of eosinophils in blood or sputum), co‐morbidities (e.g., nasal polyposis) and response to inhaled corticosteroids [1, 20]. Overall, type 2‐high asthma is closely associated with childhood‐onset, allergies, and eosinophilic inflammation, with patients showing different degrees of symptom severity and corticosteroid responsiveness [20, 24]. Strikingly, the strongest and most reproducible genetic risk variants for asthma localize near genes that play key roles in type 2 immunity [17], further emphasizing the importance of this specific inflammatory pathway for asthma pathophysiology. Type 2‐low asthma is more complex and appears linked to late‐onset disease, obesity, and neutrophilic inflammation [20], although no reliable biomarkers have been identified. Asthma phenotyping has already been implemented in daily clinical practice, with treatment choices (e.g., use of biologics in severe asthma patients) being based on validated biomarkers linked to type 2 classification status (e.g., eosinophil counts, exhaled breath nitric oxide levels) and allergy (e.g., IgE antibody levels) [25]. Hence, in‐depth phenotyping of asthma—in particular for those patients that present with severe and therapy‐resistant disease—remains a key topic of research in the field. Here, we will review how specific immune responses drive asthma pathophysiology, placing special emphasis on type 2 immunity and the lymphocytes that orchestrate chronic (type 2) inflammation—particularly in severe disease forms.

### 1.2 Type 2 Inflammation

Humans have evolved a complex immune system to neutralize pathogens and malignant cells. Innate immune cells such as macrophages or dendritic cells respond rapidly by recognizing common pathogen constituents or danger signals released by the tissue microenvironment. These cells will activate T and B lymphocytes, which use a near‐infinite repertoire of specialized receptors to launch highly specific adaptive immune responses against non‐self substances [26]. Importantly, our immune system tailors its response to the specific microbial threat it is up against: type 1 responses protect against intracellular pathogens such as viruses and bacteria, type 2 responses expel parasites and conduct tissue repair, whereas type 3 responses combat extracellular bacteria and fungi [27]. Each of these types of immunity is fueled by the activation of a distinct combination of innate lymphoid cell (ILC) and T cell subsets, which through cytokine production and cell–cell contact, orchestrate powerful immune responses. Type 1 immunity is characterized by the group 1 ILCs (“ILC1s”), T helper 1 (“Th1”) cells, and cytotoxic T (“Tc1”) cells that produce IFNγ and TNFα cytokines; type 2 immunity is driven by ILC2s, Th2 cells, and Tc2 cells that secrete the type 2 cytokines interleukin 4 (IL‐4), IL‐5, IL‐9, and IL‐13; and type 3 immunity involves ILC3s, Th17 cells, and Tc17 cells that release IL‐17 and IL‐22. These archetypical cytokines, in turn, trigger complex cascades of molecular and cellular events that culminate in tissue inflammation. Given its central role in asthma, we will primarily focus on type 2 inflammation in this review.

### 1.3 Th2 Cells as Classic Drivers of Type 2‐High Asthma

**FIGURE 1:**
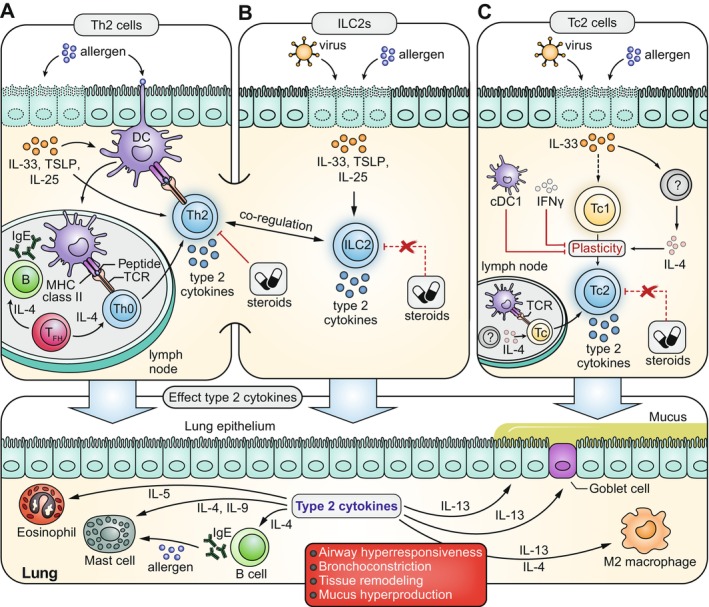
Th2 cells, ILC2s and Tc2 cells as drivers of type 2‐high asthma pathophysiology. In type 2‐high asthma, triggers such as allergens but also viral infections damage and/or activate the lung epithelial cells (indicated by dashes cell outlines), which in turn secrete alarmins such as IL‐33, TSLP, and IL‐25. (A) Alarmins can activate dendritic cells (DCs), which can take up allergens and migrate to lymph nodes. Here, antigen presentation to naive T cells via major histocompatibility (MHC) class II—T cell receptor (TCR) interactions combined with IL‐4 signals from follicular T helper (Tfh) cells induce Th2 cell differentiation. Th2 cells migrate back to the lungs and start producing type 2 cytokines IL‐4, IL‐5, IL‐9, and IL‐13, which can be (1) further stimulated by alarmins, (2) regulated by ILC2 activity or (3) efficiently suppressed by corticosteroids. (B) Viral infections and alarmins can induce rapid local secretion of type 2 cytokines by activating lung‐resident ILC2s, which can further augment Th2 responses and can adopt steroid‐resistant phenotypes. (C) IL‐33, perhaps directly (dashed arrow) and/or via the induction of local IL‐4 production, may induce Tc1‐to‐Tc2 plasticity to increase local Tc2 cell abundance and type 2 cytokine production. Skewing of the lung Tc compartment to a Tc2 phenotype can be suppressed by conventional type 1 DCs (cDC1s) and IFNγ signaling. Allergen‐specific Tc2 responses (via a DC‐mediated lymph node response, also see A) may also play a role in generating Tc2 cells in the lung. Tc cells are generally considered more resistant to corticosteroids. The bottom panel depicts the downstream cellular targets of the type 2 cytokines, ultimately resulting in the hallmark symptoms of asthma indicated in the red box.

A classic hallmark of type 2 immune responses is the emergence of eosinophilic inflammation, which is triggered by a series of signals that originate from the tissue epithelium [20]. For example, invading parasites but also inhaled allergens can damage or activate lung epithelial cells, causing the release of cytokines called “alarmins” that activate dendritic cells. Prominent alarmins include IL‐33, IL‐25, and thymic stromal lymphopoietin (TSLP). Activated dendritic cells take up parasite or allergen antigens and migrate to the lymph nodes to present these to naive CD4^+^ T cells. Additional co‐stimulation from dendritic cells combined with exposure to IL‐4 produced by follicular T helper (Tfh) cells promotes the differentiation of these naive T cells into Th2 cells, which subsequently migrate back to the lung [17, 20]. Here, Th2 cells start producing large amounts of type‐2 cytokines, which directly or indirectly control all aspects of the type‐2 immune response and induce the hallmark symptoms of asthma (Figure 1A): IL‐5 induces bone marrow eosinophil differentiation and recruitment into the lung; IL‐4 stimulates antibody class switching and IgE production by B cells; IL‐9 promotes the survival and proliferation of histamine‐producing mast cells; IL‐13 reduces the barrier function of epithelial cells and increases mucus production by goblet cells as well as smooth muscle cell hyperreactivity [17, 20]. The establishment of long‐lived circulating and lung‐resident memory Th2 cells, which retain the ability to rapidly reactivate upon repeated antigen or epithelial alarmin exposure [28, 29], further contributes to the prolonged production of large amounts of type 2 cytokines in type 2‐high asthma [30, 31]. Importantly, chronic activation of this type 2 immune response—as observed in many asthma patients—can result in persistent airway wall remodeling and mucus plugging, which is a major contributor to chronic airflow obstruction in severe asthma [32]. Since the majority of asthma patients show hallmarks of type 2 and/or eosinophilic inflammation, Th2 cells and the type 2 cytokines they produce have been a dominant topic of investigation and a major target for therapy development in the past 30 years [33, 34, 35].

### 1.4 Chronic Inflammation in Asthma: Looking Beyond the Th2 Cell

Despite the central role of Th2 cells in producing type 2 cytokines, the field has kept a keen interest in identifying other sources of IL‐4, IL‐5, IL‐9, and IL‐13, which may also be relevant for asthma pathobiology. One important argument for this is that a substantial number of severe therapy‐resistant asthma patients present with a type 2‐high phenotype or associated biomarkers (> 50%) [36, 37], despite the repeated observation that type 2 cytokine production by Th2 cells is effectively suppressed by corticosteroids [38, 39, 40, 41, 42]. This indicates that corticosteroid‐resistant producers of type 2 cytokines critically contribute to severe asthma. Moreover, the presence of substantial numbers of type 2‐low asthma patients—often showing a more severe disease course and therapy resistance [1, 20]—has sparked interest to investigate the role of non‐type 2 inflammation in asthma [43, 44]. In this review, we will therefore look beyond the Th2 cell and focus on lymphocyte subsets implicated in driving severe, therapy‐resistant disease.

## 2 Group 2 Innate Lymphoid Cells (ILC2s): The New Kid on the Block

Various alternative non‐T cell producers of type 2 cytokines have been identified throughout the years. IL‐4 can also be produced by natural killer T (NKT) cells, mast cells, and basophils [45], and our recent work has shown that IL‐33 promotes the rapid formation of large numbers of IL‐4‐producing eosinophils in the lungs of mice [46]. Similarly, IL‐5 can also be synthesized by NKT cells, mast cells, and eosinophils [47, 48], and IL‐13 secretion has been reported for B cells, macrophages, mast cells, and basophils [49]. B cells and mast cells have also been described as IL‐9 producers [50]. However, the most prominent producers of type 2 cytokines apart from Th2 cells are the ILC2s [27, 51], which were formally discovered in 2010 (in mice [52, 53, 54]) and 2011 (in humans [55, 56]). Since then, numerous studies have been launched to uncover the roles played by ILC2s in type 2 immune responses and beyond, revealing important functions for ILC2s in anti‐parasite immunity, tissue repair after viral clearance, control of metabolic and adipose tissue homeostasis, suppressing age‐associated neuroinflammation, and even anti‐tumor immunity [51, 57, 58, 59, 60]. Recent studies have implicated ILC2 dysfunction in several important causes of human morbidity, including type 2 immunopathologies such as asthma. In this section, we will review current knowledge on ILC2 function in the context of type 2 inflammation and asthma.

### 2.1 Basic ILC2 Biology

**FIGURE 2:**
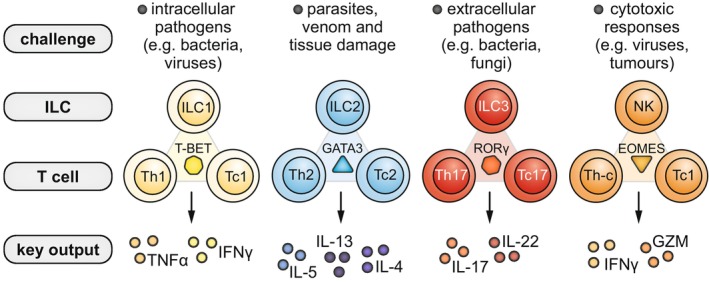
Overview of specific immune responses and key lymphocyte subsets involved. Different types of immune challenges induce responses driven by specific innate lymphoid cell (ILC), CD4^+^ T helper (Th) and CD8^+^ cytotoxic T (Tc) cell subsets. For each type of challenge, ILC and T cell counterparts rely on the same lineage‐specifying transcription factor (i.e., T‐BET, GATA3, RORγ or EOMES) to converge on the production of specific effector cytokines or granzymes (“key output”). Th‐c cells are CD4^+^ Th cells with cytotoxic properties. Tc1 cells depend on both T‐BET and EOMES.

As the ‘innate’ in their name already implies, ILCs do not carry antigen‐specific receptors required for adaptive immune responses. Instead, ILCs rely on signals from their tissue microenvironment to guide their activity patterns, including cytokines and metabolites. As mentioned above, an ILC counterpart has been identified for most major T cell subsets: ILC1s & Th1 cells, ILC2s & Th2 cells, ILC3s & Th17 cells, and NK cells & Tc1 cells [61, 62] (Figure 2). Functionally, ILCs often fulfill similar roles to T cells in various immune responses, although there are several important differences. Unlike T cells that originate from the thymus, the main site of ILC development is the bone marrow. Here, mature ILCs are generated via a differentiation process that starts from the hematopoietic stem cell and involves several progressively more restricted progenitor states, which remain poorly understood—especially for human ILCs [61, 63, 64]. Mature naive ILCs leave the bone marrow to seed peripheral tissues during fetal development, where they sustain themselves through self‐renewal as a population of tissue‐resident innate immune cells [65, 66]. Next to key sites of hematopoiesis such as the bone marrow and fetal liver, multipotent human ILC progenitors (‘ILCPs’) have been found in various (non‐)lymphoid tissues as well as in both cord and adult blood [67, 68, 69, 70], providing a potential means for replenishing ILC tissue reservoirs if needed [67, 71].

Like Th2 cells, ILC2s critically rely on the GATA3 transcription factor for their development and their ability to produce type 2 cytokines [72]. As bona fide lymphocytes, ILC2s are dependent on common γ‐chain cytokines for their development and maintenance [52, 53, 54, 73], such as IL‐15 and particularly IL‐7. Due to the absence of lineage‐defining markers (e.g., CD3 for T cells) on ILCs, distinguishing ILC2s within biological samples first requires the careful exclusion of cells expressing lineage markers and antigen‐specific receptors. Several strategies to identify ILC2s from these lineage‐negative cells have been put forward over the years, which may differ depending on tissue of origin, cell state, and species [74, 75, 76]. In general, all ILC2s express CD45 and GATA3, often—but not always—accompanied by expression of the receptors for IL‐2 (CD25) and IL‐7 (CD127). Most human ILC2s also express the prostaglandin D2 receptor (CRTH2) as well as high levels of CD161 and CD200R1 [74, 77, 78]. Receptors for cytokines that control ILC2 activity, such as for IL‐33 (ST2/IL1RL1) or IL‐25 (IL17RB), are expressed on ILC2s in a more tissue‐specific or cell state‐specific manner [74, 79, 80] and should therefore be used with caution for ILC2 identification. Although the localization of ILC2s in human lungs remains largely unexplored, mouse studies indicate that ILC2s preferentially localize near sources of alarmins—particularly epithelial cells and stromal cells [76, 81, 82, 83]. Most notable are lung adventitial cuffs containing fibroblast‐like cells producing IL‐33 and TSLP, where ILC2s and Th2 cells appear to concentrate at steady‐state [82]. Allergic insults promote local ILC2 proliferation and result in broad ILC2 as well as Th2 cell accumulation in parenchymal locations [82, 84]. However, it remains unclear whether ILC2 expansion during inflammation primarily originates from adventitial niches or whether recruitment from circulatory ILC2s and ILCPs also contributes.

Activation of tissue‐resident ILC2s does not rely on antigen recognition but occurs in response to a variety of tissue signals. Extensive in vivo studies have shown that at mucosal sites, murine ILC2s drive type 2 immune responses against extracellular parasites and allergens as well as promote tissue repair [85, 86]. Central to ILC2 activation are alarmin cytokines released by epithelial and stromal cells (Figure 1B). For example, lung tissue damage by protease allergens induces IL‐33 alarmin release, which in turn evokes the rapid production of copious amounts of IL‐5 and IL‐13 by resident ILC2s [87]. In such animal models of allergic airway inflammation, ILC2s are the major producers of IL‐9 [88], and human ILC2s can also produce IL‐9 [89, 90]. Although ILC2s are generally considered poor producers of IL‐4, both mouse and human ILC2s can secrete this cytokine under certain conditions [89, 91, 92]. Apart from the type 2 cytokines, ILC2s produce various other inflammatory and immunomodulatory mediators. These include GM‐CSF to activate dendritic cells [93], the HMGB1 alarmin to attract neutrophils [94], amphiregulin (AREG) to induce epithelial tissue repair [56], and LIF to control immune cell egress from the lungs [95]. Hence, ILC2s are largely tissue‐resident innate immune cells that control multiple aspects of type 2 immune responses.

### 2.2 Regulating ILC2 Activity

**FIGURE 3:**
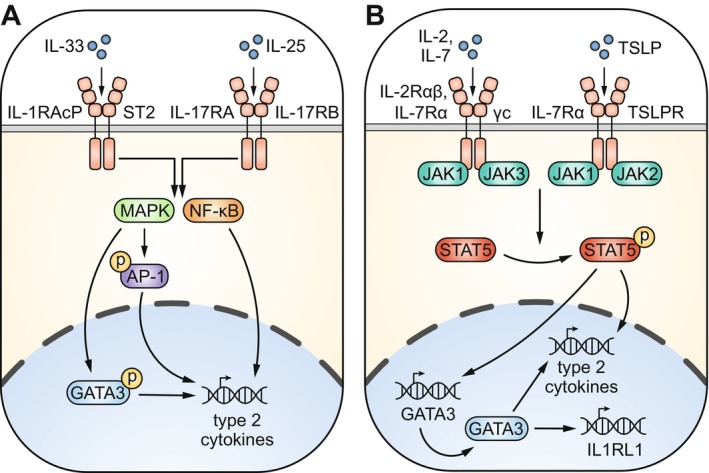
Key signaling pathways regulating ILC2 activity. (A) IL‐33 binds to a receptor heterodimer that consists of an ST2 and an IL‐1RAcP subunit. IL‐25 also binds a receptor heterodimer but instead composed of IL‐17RA and IL‐17RB. Signaling via both receptors leads to downstream activation of NF‐kB and MAPK pathways. MAPK signaling subsequently triggers AP‐1 activation, which can occur via phosphorylation (as depicted) but also through *de novo* transcription of AP‐1 family genes (not shown). As a result, NF‐κB and AP‐1 transcription factors bind DNA and promote the transcription of the type 2 cytokine genes. Additionally, MAPK proteins can induce phosphorylation of GATA3, facilitating its binding to *IL5* and *IL13* gene promoters to enhance transcription. (B) IL‐2 (IL‐2R) and IL‐7 (IL‐7R) receptor subunits pair with the common γ chain (γc). Activation of these receptor heterodimers leads to phosphorylation of the JAK1 and JAK3 kinases. JAK1/JAK3 subsequently bind and phosphorylate cytoplasmic STAT proteins—most prominently STAT5. Phosphorylated STAT5 dimerizes and translocates to the nucleus, where it acts as a transcription factor that activates the type 2 cytokine locus and but also enhances *GATA3* transcription. Elevated GATA3 levels results in further induction of type 2 cytokine gene transcription, but also promotes *IL1RL1* (encoding ST2) expression. The alarmin TSLP signals via a heterodimeric receptor consisting of TSLP receptor (TSLPR) and IL‐7R subunits. Very similar to IL‐2 and IL‐7, TSLP signals trigger a JAK‐STAT5 signaling cascade that terminates in increased transcription of the type 2 cytokine genes.

Binding of alarmin cytokines IL‐33 and IL‐25 to their receptors induces downstream intracellular signaling cascades, most notably the transcription factor nuclear factor κ B (NF‐κB) and MAPK pathways (Figure 3A). These pathways terminate into activation of NF‐κB, AP‐1, and phosphorylated GATA3 transcription factor binding to the *IL5* and *IL13* gene regulatory regions, promoting transcription of the type 2 cytokine genes [96, 97, 98, 99]. Another critical activation signal for ILC2s—akin to Th2 cells—is provided by IL‐2, IL‐7, and/or TSLP cytokines, which all trigger JAK‐mediated phosphorylation of the cytoplasmic STAT5 transcription factor [100, 101, 102, 103] (Figure 3B). Subsequent translocation of phospho‐STAT5 to the nucleus promotes *GATA3* transcription in ILC2s [89, 104, 105], and in Th2 cells, STAT5 directly binds and activates type 2 cytokines genes [106]. Of note, GATA3 and STAT5 can directly promote the transcription of key cytokine receptor genes (e.g., *Il1rl1* encoding the IL‐33 receptor) in murine ILC2s and/or Th2 cells [107, 108, 109], establishing positive feedback loops that allows for rapid and sustained type 2 cytokine production. In this context, the MEF2D transcription factor was recently identified as a critical upstream mediator that maintains the GATA3‐centered transcriptional program in mouse ILC2s and Th2 cells [110]. Interestingly, ILC2s carry receptors for effector cytokines they themselves produce. Both IL‐4 and IL‐9 have been shown to enhance inflammatory cytokine production by ILC2s, as well as promote cell survival through autocrine signaling [88, 111, 112]. Importantly, several cytokines have been reported to suppress ILC2 activity, including IL‐10 and TGFβ produced by regulatory T cells [113]. Cytokine signals associated with type 1 immunity have also emerged as potent inhibitors of ILC2s, such as IL‐12 and type I/II interferons [114, 115].

Neuropeptides and neurotransmitters can also potently modulate ILC2 activity. A prominent example is the neuropeptide neuromedin U (NMU), which signals through the NMUR1 receptor. Neurons present at mucosal barrier surfaces can produce NMU when sensing parasitic components or alarmins [116]. NMU exposure induced proliferation and enhanced type 2 cytokine production in murine ILC2s by activating downstream MAPK and NFAT pathways, promoting inflammatory responses against parasites and allergens [117, 118, 119]. Human ILC2s showed similar responses to NMU in vitro [120, 121]. NMU stimulation results in increased intracellular Ca^2+^ levels, which activates the NFAT transcription factor that then translocates to the nucleus and promotes the transcription of type 2 cytokine genes [117]. Calcitonin gene‐related peptide (CGRP) is a neuropeptide secreted by pulmonary neuroendocrine cells (PNECs), a specific type of airway epithelial cell that resides in close proximity to ILC2s. PNEC‐derived CGRP can positively regulate CGRP receptor expressing ILC2s to promote type 2 cytokine production in vivo [122]. However, other mouse studies reported CGRP as a negative regulator of ILC2 activity, as inhibition of CGRP signaling enhanced ILC2 responses [123, 124]. Combined with the lack of human studies, the precise role of CGRP in regulating ILC2 activity remains unclear. Several other neuronal signals have been shown to either activate or inhibit ILC2 function (e.g., dopamine, vasoactive intestinal peptide), as extensively reviewed elsewhere [116, 125, 126].

Lipid mediators such as leukotrienes and prostaglandins are also well‐established regulators of ILC2s activity and airway inflammation [127, 128, 129, 130]. These include prostaglandin D2 (PGD2), a potent activator of ILC2s that signals through the CRTH2 surface receptor expressed on ILC2s [131]. PDG2 can induce type 2 cytokine production (independent from alarmins), stimulate alarmin receptor upregulation, and controls ILC2 accumulation in the inflamed lung [131, 132, 133]. Leukotrienes are another class of lipid signaling molecules known to control ILC2 activity. ILC2s express the cysteinyl leukotriene receptors CysLT1 and CysLT2, and leukotriene C4 (LTC4), LTD4, and LTE4 have been shown to boost ILC2 survival and cytokine production, with IL‐4 induction appearing particularly dependent on leukotrienes [91, 134, 135]. Conversely, other lipid mediators such as PGI2, PGE2, and lipoxin A4 (LXA4) were shown to inhibit ILC2 responses [133, 136, 137], in part through the downregulation of GATA3 [136].

Beyond cytokine‐mediated regulation, ILC2 activation is also controlled through cell–cell interactions. ILC2s express many co‐stimulatory (e.g., ICOS, OX40, GITR, CD226) and co‐inhibitory (e.g., PD‐1, KLRG1, CD200R, SIRPα, SLAM family receptors) molecules [114, 127, 138, 139, 140]. Mechanistically, most of these pathways converge on modulating GATA3 levels or the activity of the alarmin signaling cascades, resulting in altered ILC2 proliferation and type 2 cytokine output. Interestingly, activated ILC2s were recently shown to upregulate the transferrin receptor to facilitate iron uptake—a nutritional trace element that is required for ILC2 proliferation and cytokine production [141]. In contrast, force and pressure sensed by the Piezo1 mechanosensitive ion channel restrains ILC2 effector functions mainly via metabolic rewiring [142]. Altogether, a large body of research has revealed that ILC2s express a myriad of receptors for receiving immunoregulatory signals from their tissue microenvironment, allowing for the fine‐tuning of ILC2 responses in a variety of immunological contexts—including eosinophilic airway inflammation.

### 2.3 ILC2 Identity and Plasticity

**FIGURE 4:**
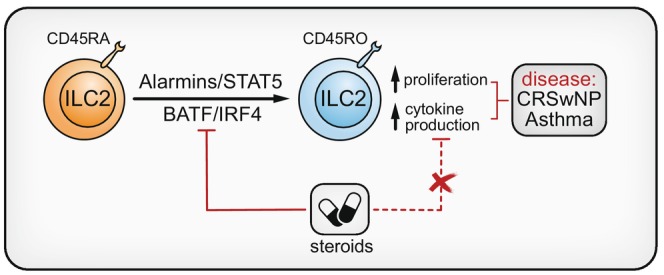
Human inflammatory CD45RO^+^ ILC2s and severe type 2 respiratory disease. Resting human ILC2s express the CD45RA splicing isoform of the CD45 surface receptor. Upon combined exposure to alarmins such as IL‐33 or IL‐1β and a STAT5‐inducing cytokine (e.g., IL‐2, IL‐7), these CD45RA^+^ ILC2s convert into CD45RO^+^ ILC2s that are highly proliferative and secrete large amounts of type 2 cytokines. This CD45RA‐to‐RO conversion is tightly linked to the upregulation of the BATF and IRF4 transcription factors and can be effectively suppressed by corticosteroids. However, once converted, CD45RO^+^ ILC2s show reduced sensitivity to corticosteroids, and their abundance positively correlates with increased disease severity in asthma and chronic rhinosinusitis with nasal polyps (CRSwNP) patient samples.

Over the past years, several distinct ILC2 subsets have been identified. In mice, natural ILC2s (nILC2s) are present at steady‐state and are tissue‐resident cells that primarily respond to IL‐33. During inflammation, a population of ILC2s with broader responsiveness (e.g., to IL‐25) marked by high levels of KLRG1 and BATF expression were found to accumulate in the lung, which were named inflammatory ILC2s (iILC2s) [143, 144, 145]. iILC2s can differentiate into nILC2(‐like) cells [143], and iILC2 emergence relies on Notch signaling [146]. Strikingly, iILC2s can display migratory behavior and enter the circulation, including extrusion from tissues after local proliferation and inter‐organ trafficking [144, 145, 147, 148]. In mouse models of helminth infections, migratory iILC2s are considered a key source of type 2 cytokines and critical for subsequent worm expulsion [144, 147]. In mice, iILC2 trafficking from the gut to the lungs can be induced by gut commensals, which promotes steady‐state pulmonary eosinophilia that shields from bacterial infections but also exacerbates allergic airway inflammation [149]. In humans, ILC2s can be detected at low frequencies (~0.025% of CD45^+^ cells) in peripheral blood [67]. We previously described an inflammatory human ILC2 population marked by CD45RO present in inflamed mucosal tissue and in peripheral blood [90]. CD45RO^+^ ILC2s can be differentiated in vitro from quiescent CD45RA^+^ ILC2s upon exposure to IL‐33 and a STAT5‐inducing cytokine (e.g., IL‐2, IL‐7), generating a highly proliferative ILC2 population that produces substantial levels of IL‐5 and IL‐13 [90] (Figure 4). Interestingly, similarities in protein expression (e.g., KLRG1+, BATF+), migratory capacity, and broad transcriptomic signatures suggest that human CD45RO^+^ ILC2s may represent a functional counterpart of mouse iILC2s [90].

Th2 cells can adopt a memory phenotype, a stable circulatory or tissue‐resident cell state that enables rapid recall responses [150]. Compared to their naïve counterparts, memory T cells show enhanced proliferation and cytokine production upon stimulation, and memory Th2 cells are considered pivotal in fueling chronic airway inflammation in asthma [35]. Although immunological memory has long been viewed as a feature unique to adaptive immune cells (i.e., T and B cells), we now know that innate immune cells can also maintain a hyperresponsive “memory‐like” phenotype after their initial activation—a concept commonly referred to as “trained immunity” [151, 152]. In 2016, Martinez‐Gonzalez et al. demonstrated that mice previously exposed to an allergen (or IL‐33) exhibited enhanced type 2 cytokine responses by lung ILC2s upon secondary allergen challenge, both in vitro and in vivo [153]. Other studies in mouse models confirmed that ILC2s can be “trained” [154], including reprogramming of their chromatin landscape to facilitate rapid inflammatory gene re‐activation [155]. Recently, a human CD45RO^+^ ILC2 subset was described with memory‐like features, including hyperresponsiveness to secondary stimulation [156]. Although the molecular mechanisms underlying the acquisition and maintenance of a trained or memory‐like state in lung ILC2s remain poorly understood, several groups have reported surface markers and transcription factors linked to a trained ILC2 phenotype. These include alarmin receptors [154, 155, 157], the ICOS co‐stimulatory molecule [155], the c‐Maf transcription factor [158], CD45RO isoform expression, and the downregulation of IL‐7 receptor expression [156].

**FIGURE 5:**
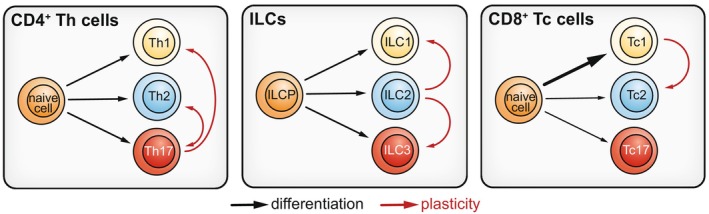
Lymphocyte differentiation and plasticity. The major differentiation routes from multi‐potent naive T helper (Th), T cytotoxic (Tc) and ILC progenitors (ILCP) are indicated by black arrows; arrow thickness in the Tc cell panel denotes that Tc1 differentiation is the dominant route. However, the identity of mature T cells and ILCs is not set in stone: microenvironmental signals such as cytokines can destabilize lymphocyte identity, resulting in the acquisition of functional characteristics (e.g., cytokine production) otherwise restricted to other subsets—a phenomenon referred to as plasticity (red arrows). Particularly plastic among lymphocytes are Th17 cells and ILC2s. Not all possible routes for plasticity are indicated; we focused on those supported by substantial evidence and of potential relevance to asthma.

The ability of differentiated effector lymphocytes to acquire functional characteristics normally linked to other lymphocyte subsets is often referred to as “plasticity”. Such phenotypic flexibility can be advantageous if microenvironmental changes demand non‐canonical responses, but can also be detrimental if plasticity‐inducing signals persist (e.g., chronic inflammation). Whereas Th1 and Th2 cell identities appear rather stable, Th17 cells exhibit a notoriously plastic nature [159] (Figure 5). In line with their diverse tissue signal receptor repertoire, ILC2s appear to have an exquisite talent for plasticity [62]. For example, mouse iILC2s—but not nILC2—express both GATA3 and RORγt (i.e., the Th17/ILC3 master transcription factor), enabling them to produce type 2 cytokines but also IL‐17 depending on which microenvironment signals they are exposed to [143]. Human ILC2s can adopt ILC1‐like or ILC3‐like phenotypes when exposed to cytokines that induce type 1 (e.g., IL‐12) or type 3 (e.g., IL‐23) responses [160, 161, 162, 163] (Figure 5). ILC2‐to‐ILC1 plasticity was associated with severe chronic inflammation in COPD and silicosis [161, 163, 164], whereas ILC2‐to‐ILC3 plasticity has been linked to chronic neutrophilic inflammation in cystic fibrosis or psoriasis [162, 165]. Additionally, human ILC2s expressing the KIT receptor exhibit RORγt upregulation, rendering these cells susceptible to type 3 polarizing cytokines for inducing IL‐17 production [165, 166]. In contrast, CD45RO^+^ inflammatory ILC2s are resistant to plasticity‐inducing signals [90], indicative of a more terminally differentiated phenotype. The mechanisms promoting the remarkably plastic nature of ILC2s are still poorly understood, although epigenomic priming of specific genes and biological pathways likely provides a key piece of the puzzle [167, 168].

### 2.4 Interplay and Redundancy Between ILC2s and Th2 Cells

Given the functional similarities between ILC2s and Th2 cells in driving type 2 immune responses, a natural question that arises is whether they may also be functionally redundant. For a long time, this question has been difficult to address due to a lack of genetic interference strategies that only target ILC2s and do not impact other immune cells—in particular, Th2 cells [86, 169]. Early efforts made use of comparisons between wildtype mice, Rag‐deficient mice lacking B and T cells, and Rag‐γc knockout mice lacking all lymphocytes (including ILCs). These studies showed that ILC2s were necessary and sufficient to drive type 2 immune responses against various (protease) allergens [87, 170, 171, 172], although some allergic airway inflammation models (e.g., HDM) may be less dependent on ILC2s [169]. Novel recombinase mouse strains, including *Nmur1*‐Cre and Boolean‐ILC2‐Cre transgenic lines, now allow for specific ILC2 targeting in immunocompetent mice and have confirmed that ILC2s play non‐redundant roles in anti‐helminth immunity but also as drivers of allergic airway inflammation [110, 173, 174]. Interestingly, the division of labor between ILC2s and Th2 cells seems to be rather dependent on the mouse model used and on the specific phase of the immune response [86, 110, 173, 174]. Whereas Th2 cell induction can occur in the absence of ILC2s and vice versa, several studies have revealed situations in which one subset is critical for the activation of the other. Th2 cells can promote pulmonary alarmin release in an IL‐4‐dependent manner, resulting in local ILC2 expansion [174]. Moreover, IL‐2 produced by Th2 cells can promote ILC2 type 2 cytokine secretion [88, 175, 176]. Conversely, IL‐4, IL‐13, and LIF produced by ILC2s can promote Th2 cell differentiation mediated by dendritic cells in lung‐draining lymph nodes [95, 135, 177, 178]. Interestingly, both mouse and human ILC2s can process and present allergen antigens to Th cells [175, 176, 179], and ILC2s can engage in MHCII‐mediated or OX40L‐mediated dialogues with Th2 cells to promote adaptive immunity against parasites [175, 180]. Important to note when considering ILC2‐Th2 interplay are the kinetics of the various type 2 immune responses. Depending on when one examines ILC2 and Th2 cell phenotypes during a specific response, the activation status and relative importance of both subsets can be quite different. During anti‐helminth responses, an overarching model of ILC2‐Th2 interplay was recently proposed [86]. Herein, alarmin‐activated ILC2s drive the initiation phase and promote the differentiation of Th2 cells, which arrive later to sustain local cytokine production and establish long‐term immunity. Thus, evidence from mouse models of type 2 immunity indicates a temporal division of labor between ILC2s and Th2 cells, with both cell types playing important roles in fueling the inflammatory type 2 response (Figure 1A,B). In contrast, we still have a very limited understanding of the relative contributions of ILC2s and Th2 cells to chronic inflammatory diseases in humans—including asthma.

### 2.5 ILC2s in Asthma

Given the prominent involvement of type 2 inflammation in most asthma patients, ILC2s were quickly considered as a potential new pathogenic effector cell in asthma pathogenesis after their formal discovery in 2010–2011. And for good reasons: the type 2 cytokines that ILC2s produce were known to play causal roles in establishing many classic hallmarks of asthma, and the epithelial alarmins that activate ILC2s had well‐established links with asthma pathogenesis [20]. By now, numerous studies have provided evidence that ILC2s may indeed play important roles in asthma [181, 182]. Many have reported increased (cytokine‐producing) ILC2 frequencies in peripheral blood, sputum, and/or bronchoalveolar lavage (BAL) samples from asthma patients as compared to healthy donors [90, 183, 184, 185, 186, 187, 188]. Increased ILC2 abundance and/or activity was often—although not in every study—found associated with more severe symptoms, elevated exacerbation frequencies, and therapy‐resistant asthma phenotypes [20]. Notably, these changes in peripheral blood ILC2s were most pronounced in female severe asthmatics [189], which can be explained by sex hormone‐mediated regulation of ILC2 activity [190] and may explain why asthma prevalence increases in women after puberty [191]. ILC2s isolated from severe asthma patients showed increased proliferation and cytokine secretion after stimulation as compared to cells from healthy controls or mild asthmatics [192]. Allergen challenge studies in allergic asthmatics revealed increased numbers of activated ILC2s in the airways and fewer ILC2s in blood shortly (within 7 h) after exposure [121, 193, 194, 195], suggesting active trafficking of ILC2s from the circulation to the airways during pathological pulmonary type 2 inflammation. A role for ILC2s in mediating asthma risk early in life is supported by strong pulmonary ILC2 expansion in young mice [196, 197], and prenatal inflammation—a known risk factor for asthma [198, 199]—induced murine ILC2 hyperactivation and aggravated type 2 lung inflammation during adulthood [200]. ILC2s have also been implicated in virus‐induced asthma exacerbations. We have shown that when mice on a chronic HDM protocol are infected with influenza, ILC2s contribute substantially to type 2 cytokine production after viral clearance [83]. Viral challenge studies in mice and humans confirmed increased alarmin and ILC2 levels after infection, correlating with elevated type 2 cytokine production [201, 202, 203]. Finally, we have shown that common genetic variants that confer increased risk of asthma susceptibility are closely linked to ILC2‐specific transcriptional control of key asthma genes [168]. Together, these findings provide a solid basis for ILC2s as drivers of (severe) asthma.

We recently reported that CD45RO^+^ inflammatory ILC2s are elevated in the blood and inflamed mucosal tissues of patients with asthma or chronic rhinosinusitis with nasal polyps [90] (Figure 4), a common upper airway type 2 inflammatory disease and major co‐morbidity of asthma. In our cohort of asthmatics, CD45RO^+^ ILC2 abundance peaked in patients with the highest symptom burden and exacerbation frequency. Moreover, we showed that CD45RO^+^ ILC2 levels further increase during an asthma exacerbation [90]. Increased frequencies of circulating CD45RO^+^ ILC2s were also found in obese individuals with asthma as compared to non‐obese patients and (non‐)obese controls [187]. Additionally, high baseline CD45RO^+^ ILC2 levels predicted rapid and favorable responses to anti‐IL4Rα blocking antibody therapy (i.e., a biologic called dupilumab) in severe asthma patients [204]. Interestingly, cigarette smoke—known to exacerbate asthma symptoms—appears to induce CD45RO^+^ ILC3s prone to express IL‐17 [205, 206]. IL‐17 can trigger neutrophilic inflammation, which is associated with type 2‐low asthma and poor responses to corticosteroid therapy. The origin of these CD45RO^+^ ILC3s is unclear, but future studies should take the plasticity of ILC2s—in particular KIT+ ILC2s [165, 166, 207]—towards an ILC3‐like phenotype into account. CD45RO is a well‐known marker for memory T cells, and human CD45RO^+^ ILC2s with memory‐like characteristics were recently described [156]. Although it remains unclear how relevant “memory ILC2s” are in the pathophysiology of asthma, the chronic nature of asthma and the reported associations of CD45RO^+^ ILC2s with a severe disease course warrant further investigation into this topic.

### 2.6 ILC2s and Asthma Treatment

Approximately 5%–10% of asthma patients present with a form of corticosteroid‐resistant disease, leading to uncontrolled symptoms and substantial morbidity. In principle, corticosteroids can suppress type 2 cytokine production and proliferation as well as induce apoptosis in both mouse and human ILC2s [181, 190]. However, several studies have reported that ILC2s can adopt a steroid‐resistant phenotype under certain inflammatory conditions, raising the possibility that aberrant ILC2 activation drives therapy resistance in asthma. In mouse models and in vitro cultures of human ILC2s, IL‐33, and TSLP could induce corticosteroid resistance in ILC2s [42, 90, 208, 209, 210, 211]. We showed that human CD45RO^+^ ILC2s—but not CD45RA^+^ cells—are largely resistant to steroid‐mediated inhibition of type 2 cytokine production and proliferation, in line with their increased frequencies in inflamed nasal tissue and blood from therapy‐refractory type 2 respiratory disease patients [90] (Figure 4). Mechanistically, signaling via the JAK3‐STAT5 axis downstream of the IL‐2, IL‐7, and TSLP receptors appears critical for promoting corticosteroid insensitivity in ILC2s, and targeting these molecules restores ILC2 sensitivity to corticosteroids [42, 208, 210]. How exactly these signals render ILC2s resistant to steroid‐mediated suppression is largely unknown, although several studies have implicated the upregulation of anti‐apoptotic factors (e.g., Bcl‐XL) and downregulation of pro‐apoptotic factors (e.g., BAX) in this process [208, 209, 211]. Our own analysis of genes positively correlating with CD45RO^+^ ILC2 abundance under various conditions implicated cytokine‐mediated metabolic reprogramming of human ILC2s, involving activation of glutathione metabolism, as a potential mechanism underlying corticosteroid resistance [90]. Of note, Th2 cells in these studies often remained highly sensitive to steroid‐mediated inhibition, further supporting an important role for ILC2s in corticosteroid‐resistant asthma.

To treat severe corticosteroid‐resistant asthma, several biologics have been developed that target the type 2 cytokines, alarmins, or their receptors [4, 25]. These treatments can successfully reduce disease burden in many of these patients, and recent studies have begun to assess the impact of biologics on ILC2 activity in asthma. ILC2s express the IL‐4 receptor and show upregulation of IL4R signaling pathway genes in asthmatics [212]. In vitro studies using human ILC2s showed that IL‐4 can enhance ILC2 function through autocrine regulation [213]. Indeed, severe asthma patients treated with the anti‐IL4Rα antagonist dupilumab showed reduced numbers of circulating ILC2s, which contained lower levels of type 2 cytokine mRNA [213]. Studies in patients with severe chronic rhinosinusitis with nasal polyps reported similar findings [204]. Notably, cultured CD45RO^+^ ILC2s showed reduced IL‐5 and IL‐13 production in the presence of dupilumab, although dupilumab did not inhibit the generation of CD45RO^+^ ILC2s upon alarmin stimulation [204]. Human ILC2s do not express the IL‐5 receptor [214]. However, blood ILC2s from severe asthma patients treated with mepolizumab, an anti‐IL‐5 biologic, exhibited reduced receptor expression for IL‐7 (i.e., CD127), TSLP, IL‐25 (i.e., IL‐17RB) and PGD2 (i.e., CRTH2) [192]. Alarmin stimulation of ILC2s from patients treated with mepolizumab showed reduced proliferation and expression of GATA3 as well as NFATc1 [192]. Interestingly, such effects were not observed for ILC2s obtained from asthma patients receiving the anti‐IgE antibody omalizumab [192]. Thus, anti‐IL4Rα and anti‐IL‐5 therapies impact ILC2 physiology—either directly or indirectly—and are able to suppress ILC2 activity in severe type 2 respiratory disease patients, which is associated with increased therapy efficacy.

## 3 CD8 ^+^ T Cells in Asthma: Innocent Bystanders or Drivers of Type 2 Inflammation?

Most researchers in the field of type‐2 immunity and asthma have focused on investigating Th2 cells and ILC2s, since these lymphocytes are regarded as the canonical producers of type‐2 cytokines. In contrast, CD8^+^ T cells have received little attention. Classically, CD8^+^ T cells exhibit a cytotoxic T (or “Tc”) cell phenotype geared towards secreting granzymes and perforins to lyse cells infected by bacteria or viruses. Upon their activation, CD8^+^ T cells were long considered to only produce type‐1 cytokines such as IFNγ and TNFα—in line with their important defense function against intracellular pathogens. However, the spectrum of cytokine‐producing phenotypes known from the Th cells also exists among Tc cells: whereas most CD8^+^ T cells adopt a canonical IFNγ‐producing Tc1 identity, certain environmental conditions can induce type 2 (Tc2) or type 3 (Tc17) cytokine production capacity in CD8^+^ T cells (Figure 2). Together with the critical role of respiratory infections in triggering exacerbations, these observations raise the question of whether CD8^+^ T cells play a role in asthma. In this section, we will discuss the potential importance of the Tc2 cell compartment for pulmonary type 2 inflammation and asthma.

### 3.1 Tc2 Cells in Severe Asthma

The presentation of respiratory viral antigens to naive CD8^+^ T cells by dendritic cells in the lung‐draining lymph node induces the differentiation of virus‐specific Tc1 cells. Activated Tc1 cells migrate to the lung, where they induce apoptosis of virus‐infected cells through the release of inflammatory cytokines and cytotoxic molecules [215]. Following viral clearance, a small subset of virus‐specific Tc cells persists as either circulating or tissue‐resident memory cells that provide rapid responses in case of secondary infections [216]. Respiratory viral infections are thus accompanied by a large‐scale generation of effector Tc1 cells and the formation of lung‐resident Tc memory cells. Interestingly, Tc cells carry receptors for critical inducers of type 2 inflammation, particularly IL‐33 and IL‐4 [217, 218]. IL‐33 promotes Tc‐mediated antiviral responses [217], and IL‐4 was shown to phenotypically skew Tc cells from a Tc1 to a Tc2 phenotype [218, 219]. These aspects of Tc biology, combined with evidence of reduced sensitivity to corticosteroids [220], have raised interest in a potential role for Tc cells as mediators of severe (type 2‐high) asthma and exacerbations [221, 222]. Several observations in asthma patients support this view, including associations between blood or lung Tc2 levels and lung function decline, poor symptom control, and even death [223, 224, 225, 226, 227, 228, 229]. The reported reduced sensitivity of Tc cells to suppression by corticosteroids [230] is in line with a possible role for Tc2 cells in therapy‐resistant asthma. Interestingly, a recent study reported increased levels of circulating terminally differentiated memory (“TEMRA”) Tc cells specifically in asthma patients with obesity [187]—a disease endotype associated with poor therapy response [231]. However, this study did not report cytokine production profiles, leaving the functional identity of these TEMRA Tc cells unclear. Importantly, studies using mouse models of type 2‐high asthma have shown that perturbing Tc responses, either using antibody‐mediated depletion or through genetic approaches, can result in diminished eosinophilic inflammation and airway hyperresponsiveness [219, 232, 233, 234, 235, 236].

We recently set out to resolve outstanding questions regarding the role of Tc2 cells in asthma, placing special emphasis on their association with hallmarks of severe disease and on the micro‐environmental signals that govern the Tc2 phenotype [46]. In‐depth immuno‐phenotyping of the circulating T cell compartment in a clinically well‐characterized cohort of asthma patients confirmed an increased presence of Tc2 and Th2 cells in asthmatics compared to healthy controls. This difference was largely driven by patients with high symptom burden and frequent exacerbations, despite receiving a high steroid dose. Tc2 levels, particularly IL‐5+ and IL‐9+ Tc cells, peaked during asthma exacerbations at ~25% of circulating Tc cells, rivalling the quantitative increases observed for Th cells [46]. Using various mouse models and transgenic animals, we showed that transferred Tc2 cells can induce pulmonary eosinophilia, and that Tc2 generation in vivo is driven by the concerted actions of IL‐4 and IL‐33 [46]. Indeed, targeting the IL‐33 signaling pathway using blocking antibodies ameliorated exacerbation symptom severity in mouse models of type 2‐high asthma [237, 238]. We envision that an interplay between a chronic type 2‐high inflamed microenvironment and respiratory viruses, which can induce the release of epithelial IL‐33 [202, 237], promotes local as well as systemic Tc2 cell formation (Figure 1C). This elevated Tc2 cell presence provides a potent additional source of type 2 cytokine production, explaining the acute worsening of asthma symptoms during respiratory infections.

### 3.2 The Origin and Specificity of Tc2 Cells in Asthma

Nevertheless, several key aspects of Tc2 biology in asthma remain unclear. First is the origin of the expanded Tc2 compartment observed in (severe) asthma patients and mouse models. Although they represent a small proportion of the total Tc cell compartment, Tc2 cells can be readily detected in peripheral blood from healthy individuals [46, 228]. Hence, type‐2 cytokine production capacity appears to be an intrinsic feature of certain Tc cells at steady state, and increased Tc2 levels may result from the expansion of this existing pool. Additionally, cells with a Tc1 phenotype may show plasticity toward a Tc2 phenotype. The latter scenario is supported by findings from mouse studies, showing that IFNγ‐producing Tc1 cells could switch to type‐2 cytokine production when exposed to IL‐4 through the upregulation of GATA3 [46, 218, 219, 239]. Our recent finding of increased levels of Tc cells displaying a hybrid Tc1/Tc2 phenotype (i.e., producing both IFNγ and type‐2 cytokines) in exacerbating asthma patients and in mice with type 2‐high airway inflammation is in line with Tc1‐to‐Tc2 plasticity [46] (Figures 1C and 5). However, whether human (memory) Tc1 cells actually undergo bona fide conversion to a Tc2 cell state, possibly via a hybrid intermediate phenotype that involves GATA3 induction, remains to be experimentally proven. A second open question regards the specificity of Tc2 cells in type‐2 responses. It remains poorly understood whether Tc2 expansion is driven by Tc cells recognizing specific antigens. Dendritic cells may present allergen antigens to Tc cells via their cross‐presentation pathway, in line with an early study reporting Tc cells that recognize the Der‐p1 antigen from HDM in allergic skin disease patients [240]. Although evidence in asthma is scarce, our work using genetically engineered Tc cells specific for the ovalbumin (OVA) model allergen did reveal differentiation of naïve Tc cells toward Tc2 cells in lung‐draining lymph nodes after airway exposure to OVA [46]. Alternatively, Tc2 formation occurs in the absence of antigen‐specific T cell receptor stimulation, as supported by the strong Tc2 expansion in our IL‐33‐induced airway inflammation model. However, providing a T cell receptor stimulus (OVA) together with IL‐33 did boost Tc2 cell formation [46]. A recent comparison of T cell receptor repertoires in circulating human Tc1 and Tc2 cells revealed highly polyclonal repertoires for both subsets, which were largely unique—although shared clonotypes were detected (~2%) [241]. Both scenarios of antigen‐dependent and antigen‐independent Tc2 cell generation thus remain plausible and do not seem mutually exclusive.

Even though we identified IL‐33 as a critical driver of Tc2 cell formation, our in vitro studies indicate that IL‐33 combined with T cell receptor stimulation is not sufficient to drive differentiation of naive Tc cells towards a Tc2 cell phenotype. Instead, IL‐4 was able to induce GATA3 and type 2 cytokine production [46]. Surprisingly, co‐treatment with IL‐4 and IL‐33 did not further enhance Tc2 differentiation induced by IL‐4 alone, despite robust induction of IL‐33 receptor expression. These data imply that the strong effect of IL‐33 on Tc2 formation in vivo cannot be explained by a direct IL‐33 receptor‐mediated mechanism. The most plausible alternative scenario is that IL‐33 indirectly promotes type‐2 skewing of Tc cells by altering the lung microenvironment, likely by promoting local IL‐4 release that subsequently induces type‐2 cytokine production in Tc cells by increasing GATA3 levels. Indeed, exposing mice to IL‐33 evoked a widespread induction of IL‐4 production in various pulmonary immune cell types, including Th2 cells but also basophils and eosinophils [46]. Future studies are needed to test this hypothesis and identify the key source(s) of IL‐4 and IL‐33 that together promote Tc2 formation. IL‐4 produced by Th2 cells [174], ILC2s [135], basophils [112, 242] and eosinophils [163, 243, 244] has indeed been reported to promote differentiation and activation of type 2 lymphocytes. Moreover, Tc2 cells themselves can produce IL‐4, potentially forming a positive feedback loop [245, 246].

## 4 Non‐Type 2 Lymphocytes in Asthma: Key Players in Type 2‐Low Inflammation?

Many asthma patients do not show signs of type 2 inflammation or eosinophilia, and this form of type 2‐low asthma is often more difficult to treat [20]. Past investigations have implicated neutrophilic inflammation and inflammasome activation as hallmarks of such type 2‐low asthma [43, 44, 247]. Neutrophil activation mostly occurs downstream of type 1 and type 3 immune responses through the action of IFNγ, TNFα and IL‐17 cytokines [20, 248]. Th17 cells are resistant to suppression by corticosteroids [38, 249], which has resulted in an interest in understanding the potential role of type 3 inflammation in severe asthma [35, 250]. In this section of the review, we will highlight the involvement of Th1 and Th17 cells in asthma, including how dendritic cell subsets promote their differentiation. Moreover, we will discuss links between IL‐17 producing ILCs and asthma.

### 4.1 Th1 and Th17 Cells in Asthma

Type 1 cytokines such as IFNγ—produced by Th1 and Tc1 cells—have classically been viewed as suppressors of type 2 inflammation by antagonizing Th2, Tc2, and ILC2 cell activity [46, 84, 251, 252, 253, 254, 255]. In support, mice with a targeted deletion of T‐bet, the key transcription factor orchestrating Th1 cell differentiation, spontaneously develop an eosinophilic asthma phenotype [256]. However, murine Th1 cells have the capacity to enhance the activation or recruitment of Th2 cells to the airways [257, 258, 259]. Moreover, neutralization of IFNγ production significantly suppressed airway inflammation in vivo [260, 261]. In patients, pulmonary IFNγ responses were enhanced in severe asthma associated with neutrophilic inflammation, and serum IFNγ levels correlated with airway responsiveness [262, 263, 264, 265]. Muehling et al. observed an exaggerated Th1 responses to rhinovirus in allergic asthma patients, which may promote disease even after the infection has resolved [266]. Other studies reported that patients with allergic asthma display defective type 1 responses to rhinoviruses, including a shift towards a type 2 phenotype [267, 268]. Also, childhood non‐allergic obesity‐related asthma has been associated with systemic Th1 polarization that correlates with pulmonary function deficits [269]. However, administration of nebulized recombinant IFNγ or subcutaneous human IL‐12 to asthma patients did not improve disease symptoms [264, 270]. Taken together, Th1 cells appear to have the ability to both inhibit and enhance the activity of type 2 cytokine‐producing lymphocytes in asthma, with links to neutrophilic inflammation and differential roles in stable versus exacerbating asthma patients. Genetic variation across patients or differences between humans and mice may contribute to the reported discordant effects of Th1 cell activation. It is also conceivable that Th1 cells play different roles at various stages of the inflammatory response, for example, in the initiation phase, in established asthma, or during asthma exacerbations, with paralleling findings for Th17 cells described below [271]. In this context, it is of note that Th1 cells may show plasticity towards the Th2 lineage, that is, a hybrid Th1/Th2 phenotype, dependent on environmental signals that drive epigenetic changes [159, 272].

Since the identification in 2005 of a distinct IL‐17‐producing T cell subset [273, 274], evidence is accumulating for a role of Th17 cells in type 2‐low asthma. IL‐17 can recruit neutrophils into the airway via chemokine release from bronchial epithelial cells, particularly CXCL8 (also known as IL‐8) [275]. Conversely, neutrophil extracellular traps (NETs) and cytoplasts (enucleated cell bodies) directly activate T cells and specifically enhance Th17 cell differentiation [276, 277]. Several lines of evidence support a role for NETs and IL‐17 in severe asthma. Th17 cells and IL‐17A levels are increased in asthma, particularly in the airways of patients with neutrophil or severe therapy‐resistant asthma [278, 279, 280, 281, 282, 283, 284]. BAL fluid from patients with severe neutrophilic asthma had detectable NETs and neutrophil cytoplasts, which positively correlated with IL‐17 concentrations in BAL fluid [276]. High levels of extracellular DNA and soluble NET components in sputum mark a subset of patients with more severe asthma that display markers of inflammasome activation in their airways [43]. In this context, it is of note that corticosteroids inhibit neutrophil apoptosis and enhance Th17 differentiation (reviewed in Xie et al. [41]). Moreover, IL‐17 and IL‐23 have the capacity to inhibit glucocorticoid receptor‐α (GRα) activity and to upregulate GRβ, which can act as a dominant negative regulator of GRα. Very recently, it was reported that the IL‐23/Th17 axis suppresses a specific subpopulation of CD39^+^CD9^+^ interstitial macrophages that have the capacity to suppress NET formation and Th17 activation in mouse models. This is particularly relevant, given that decreased pulmonary CD39^+^CD9^+^ interstitial macrophages abundance was observed in patients with severe neutrophilic asthma [285]. Next to Th17 cells, other cell types also have the ability to produce IL‐17, including γδ T cells, CD8^+^ T cells, B cells, and ILC3 (see below).

The neutrophil‐IL‐17 axis can also promote type 2 immune responses in asthma. Mechanistically, it was demonstrated in mice that LPS‐induced neutrophils and NETs potentiated allergen uptake by dendritic cells and HDM‐induced allergic airway inflammation [286]. Moreover, IL‐17 signaling‐deficient mice displayed markedly reduced eosinophilia, IgE levels, and reduced Th2 cytokine production in an OVA‐driven allergic airway inflammation model [271, 287]. In contrast, IL‐17 can reduce symptoms of established allergic asthma by inhibiting DC function and chemokine synthesis [271, 287]. Interestingly, hybrid Th2/Th17 cells expressing both IL‐4 and IL‐17 were identified in BAL fluid and blood from patients with severe asthma, correlating with eosinophil counts and IgE levels [288, 289, 290] (Figure 5). Antigen‐specific IL‐17+ Th2 cells induced a profound influx of both eosinophils and neutrophils and exacerbated asthma in mice. In apparent contrast, Choy et al. observed a negative correlation between the expression of Th2‐ and Th17‐associated gene signatures in bronchial biopsies of 51 asthma patients [291].

Taken together, several lines of evidence support a role for IFNγ and IL‐17 in type 2‐low asthma. However, neutrophilic asthma is not always dependent on type 1 or type 3 responses [292], and type 2 responses can be associated with pulmonary neutrophilia. For example, ILC2s can produce the HMGB1 alarmin to initiate neutrophil chemotaxis [94]. To add to this complexity, IFNγ and IL‐17 may also act as endogenous regulators of type 2‐high asthma, modulating the inflammatory landscape and disease severity. Hence, it may not be very surprising that clinical trials with anti‐IL‐17 antibodies have thus far failed to show treatment effect [293]. However, improved patient selection (i.e., based on sputum neutrophil counts) and combinations with anti‐type 2 cytokine biologics seem worth exploring in future studies.

### 4.2 GATA3 Levels and Th Cell Differentiation

Dissection of the mechanisms involved in Th17‐dependent airway inflammation is complicated by the extensive plasticity of the Th17 lineage [159] (Figure 5). Early *Gata3* deletion in an experimental autoimmune encephalomyelitis (EAE) mouse model resulted in a defect in the generation of pathogenic T‐bet expressing Th17 cells. However, late *Gata3* deletion did not affect the generation of these T‐bet^+^ Th17 cells, although they failed to induce EAE symptoms due to reduced GM‐CSF production [294]. On the other hand, we found that transgenic mice with enforced expression of *Gata3* in the T cell lineage (via a CD2‐Gata3 transgene) were not susceptible to EAE [295]. Despite the normal numbers of Th17 cells generated, we observed concomitant production of IL‐4 and IL‐10 in Th17 cells, which likely constrained Th17‐mediated pathology. The CD2‐Gata3 transgenic mice did show increased formation of IL‐33R‐expressing Th2 cells [109]. Moreover, enforced *Gata3* expression was sufficient to enhance Th2 and ILC2 activity, leading to increased susceptibility to allergic airway inflammation after mild allergen exposure that otherwise induced tolerance [296]. GATA3 represses Th1 differentiation and Th1 cells express GATA3 only at low levels, its binding sequestered away from Th2‐specific genes [297]. Accordingly, *Gata3* overexpression inhibited Th1 cell differentiation—both in vitro and in vivo [109]. Hence, the role of GATA3 in shaping the Th1/Th2 fate decision is well‐defined, whereas the impact of GATA3 activity on Th17 cell differentiation is complex and remains poorly understood.

### 4.3 Dendritic Cell Subsets and T Cell Fate Decisions in Asthma

Differentiation of naive CD4^+^ T cells to effector cells, including Th1, Th2, and Th17 cells, is driven by dendritic cells [298] (Figure 1A). Conventional dendritic cells (cDCs) are derived from precursors in the bone marrow and comprise two subsets. Whereas cDC1s are mainly required for the induction of Tc cell activity by cross‐presentation of tumor and viral antigens, cDC2s initiate the activation and differentiation of naive CD4^+^ T cells into Th subsets. Hereby, the cytokine milieu plays a decisive role. IL‐12 is crucial for the polarization into Th1 cells. Differentiation of Th2 cells depends on low IL‐12 and high IL‐4 levels, the latter provided by follicular T helper cells in lung‐draining lymph nodes [299, 300], which promote expression of GATA3. The Th2‐polarizing capacity of cDCs is further supported by (i) low antigen dose, (ii) TSLP and IL‐33 alarmins produced by activated epithelial cells, and (iii) ILC2‐derived IL‐13, which is critical for eliciting production of the Th2 cell‐attracting chemokine CCL17 by cDC2s [178, 301, 302]. Th17 cell differentiation involves the TGF‐β, IL‐6, IL‐1β, and IL‐23 cytokines [159].

Migratory cDC2s are the principal subset that induces allergen‐specific Th2 cells in the lung‐draining lymph node of mice with HDM‐driven allergic airway inflammation [303]. By contrast, monocyte‐derived DCs (moDCs) are poorly migratory cells that mainly produce proinflammatory chemokines to maintain allergic inflammation in the lung. Stimulation of DCs by pattern recognition receptors (e.g., TLRs) induces the production of pro‐inflammatory cytokines via activation of NF‐κB. Hereby, the TNFα‐induced protein 3 (TNFAIP3; also known as A20), which controls the levels of several key intermediate NF‐κB signaling molecules, acts as a brake on cDC activation. Targeted deletion of TNFAIP3 triggers spontaneous DC maturation and hyperresponsiveness to activating stimuli, providing a valuable tool to study the role of cDCs in allergic airway inflammation [304]. Deleting *Tnfaip3* in the majority of macrophages, monocytes, neutrophils, and cDCs as well as moDCs resulted in Th1/Th17‐driven neutrophilic airway inflammation in a HDM model of asthma [305]. Likewise, specific deletion of *Tnfaip3* in all dendritic cell subsets and alveolar macrophages induced resistance to the development of eosinophilic inflammation and promoted potent lung neutrophilia [304]. The capacity of *Tnfaip3*‐deficient cDCs to induced HDM‐specific Th17 cell differentiation could be explained by the strong presence of IL‐1β, IL‐6, and IL‐23 [304]. Concomitantly, Th2 cell differentiation was hampered by increased IL‐12 and IL‐6 production. Interestingly, we showed that HDM‐driven neutrophilic airway inflammation in mice carrying *Tnfaip3*‐deficient cDCs was independent of IL‐17, supporting the concept that neutrophilic airway inflammation in asthma models can be IL‐17‐independent and may involve Th1 cells or HMGB1‐producing ILC2s. Although a role for cDC1s in asthma remains controversial, it is generally believed that cDC1s limit type 2 responses via IL‐12 production. Consistent with this notion, we found that mice harboring hyperresponsive *Tnfaip3*‐deficient cDC1s did not develop Th2‐driven eosinophilic airway inflammation upon HDM exposure [306]. Instead, these animals showed elevated numbers of IFNγ‐expressing Th1 and Tc1 cells in BAL fluid. Blocking either IL‐12 or IFNγ restored Th2 responses and eosinophilia. Moreover, we recently showed that uncontrolled *Tnfaip3*‐deficient cDC1 activity suppressed Tc2 cell formation via IFNγ in a HDM‐driven asthma model [46]. Hence, the activation status of pulmonary cDC1s may critically control the development of type 2 airway inflammation via the IL‐12/IFNγ axis. In line with these findings in mouse models, we observed that in asthma patients circulating cDC1s are relatively decreased [307]. In contrast, blood cDC2 of asthma patients with frequent exacerbations are relatively increased and display an aberrant phenotype characterized by increased CD86 and PD‐L1 expression on the cell surface.

### 4.4 IL‐17 Producing ILCs and Asthma

ILC3s represent another major source of IL‐17. In a mouse model of obesity‐associated asthma, airway hyperreactivity—a cardinal feature of asthma—was dependent on IL‐17 producing ILC3s that were activated by macrophage‐derived IL‐1β [308]. Findings from another in vivo study support a potential key role for ILC3s in obesity‐associated asthma [309]. IL‐17+ ILCs resembling ILC3s were also detected in BAL samples from (severe) asthma patients [207, 308]. A study in asthmatic children reported increased ILC3 frequencies in peripheral blood from obese as compared to non‐obese patients [310], although this appeared independent from blood neutrophil/eosinophil counts, elevated IL‐17 RNA levels, and increased disease severity. In contrast, others have shown that ILC3s can limit allergen‐specific adaptive immune responses in mice [311].

Another source of ILC‐derived IL‐17 are ILC2s that show phenotypic plasticity upon (chronic) exposure to type 3 inducing signals (see above) (Figure 5). In this context, human KIT+ ILC2s may be of particular relevance given their intrinsic propensity to readily adopt an ILC3‐like identity [165, 166]. Increased levels of the KIT ligand, stem cell factor (SCF), were found in the serum of asthma patients as well as in the lungs of mice with allergic airway inflammation [312], with one study reporting SCF levels positively correlating with asthma severity [313]. However, SCF exposure mainly augmented type 2 cytokine production in both mouse and human ILC2s, and did not induce IL‐17 production [166, 312]. Interestingly, Ju et al. recently provided evidence that ILC2‐to‐ILC3 plasticity may indeed be relevant for severe asthma [207]. They showed increased levels of IL‐17+KIT+ ILC2s in the sputum of severe asthma patients, which correlated with neutrophilic inflammation [207]. In this study, ILC2‐to‐ILC3 plasticity appeared to be mostly driven by IL‐1β and IL‐18, although the authors did not investigate the role of SCF. Together, these studies support a role for ILC3s—either bona fide ILC3s or ILC3‐like ILC2s—in severe asthma, warranting further investigation.

## 5 Future Directions

The Th2 cell has long been recognized as a central player in asthma. However, it has now become clear that other type 2 and non‐type 2 lymphocytes, such as ILC2s, Tc2 cells, and Th17 cells, play key roles in driving chronic allergic lung inflammation in mouse models and in patients suffering from asthma. Importantly, these subsets have been associated with uncontrolled asthma and type 2‐low asthma, a patient group that represents the major clinical unmet need. Therefore, non‐Th2 lymphocytes will need to constitute a primary focus of future asthma research.

Tc2 cells, Th17 cells, and ILC2s have been reported to adopt corticosteroid‐resistant states [41, 90, 114, 208, 210, 221, 314]. We have shown that the presence of Tc2 cells and CD45RO^+^ ILC2s in the blood of asthma patients is indicative of corticosteroid resistance, a severe symptom load, and higher exacerbation frequencies [46, 90]. IL‐33 plays a central role in the generation of both these cell types, indicating that biologics targeted at this alarmin [315] may be particularly useful in uncontrolled asthma patients with profound Tc2 cell and/or CD45RO^+^ ILC2 activity. Other critical activation signals are TSLP (for CD45RO^+^ ILC2s) and IL‐4 (for Tc2 cells), which can both be effectively targeted using currently approved biologics [25, 316]. Given the critical role of GATA3 as a broad master regulator of Th2, Tc2, and ILC2 differentiation, specific inhibition of this factor may also hold promise [317, 318, 319]. Apart from these canonical targets, future studies into the mechanisms that support aberrant Tc2 and (CD45RO^+^) ILC2 activity in asthma have the potential to identify novel therapeutic strategies specifically aimed at suppressing these lymphocyte subsets.

A critical aspect of lymphocyte biology is their ability to undergo phenotypic plasticity when confronted with changing microenvironmental signals. Lymphocyte plasticity is potentially highly relevant for asthma pathophysiology, as illustrated by our work providing evidence for Tc1‐to‐Tc2 skewing in the asthmatic lung [46]. Moreover, ILC2s appear exquisitely susceptible to phenotypic adaptation in a setting of chronic inflammation. Elegant work in mouse models and cultured human cells has started to tease apart the mechanisms underlying such plasticity, although we still have limited data from lymphocytes obtained from the airways of asthmatics. Recent efforts to generate human lung cell atlases at the level of single cell transcriptomes have provided a major first step in that direction and are expected to yield novel candidates for therapeutic targeting in asthma patients. However, plasticity and phenotypic flexibility are anchored in a cell's epigenome [29, 159, 320], thus calling for multi‐omics approaches that allow simultaneous measurement of epigenetic changes and gene expression dynamics in the same cell [321]. Moreover, longitudinal analyses to capture the dynamics of cellular identities throughout the inflammatory cascade—for example, through human challenge studies—will be critical to capture transitions in lymphocyte cell states and link these to specific pathophysiological aspects. For instance, time‐course lung sampling in asthma patients challenged with rhinovirus will provide unique opportunities to study the adaptation of anti‐viral responses (e.g., Tc1 cells) to chronic type 2 inflammation (e.g., Th2 cells and ILC2s) and uncover why respiratory infections exacerbate asthma symptoms.

Another major knowledge gap is our lack of systematic insight into how T cells and ILCs are spatially organized in the human lung and how this localization is altered in (severe) asthma. Given the recent breakthroughs in high‐dimensional spatial analysis methods of tissues (e.g., spatial transcriptomics [322]), we expect major new insights into the spatial principles and cellular crosstalk underlying chronic inflammation in the asthmatic lung to emerge in the coming years. This information will be instrumental for translating new developments from mouse models to asthma patients, such as the role of neuro‐immune circuits in shaping type 2 immunity.

## Conflicts of Interest

The authors declare no conflicts of interest.

## Data Availability

The authors have nothing to report.
